# Supplementary figures and images for: Living upside down: patterns of red coral settlement in a cave
Source: PeerJ. 2018 May 21;6:e4649. doi: 10.7717/peerj.4649 (PMC5967367; doi:10.7717/peerj.4649)

$$\text{DeltaK} = \text{mean}(|L''(K)|) / \text{sd}(L(K))$$

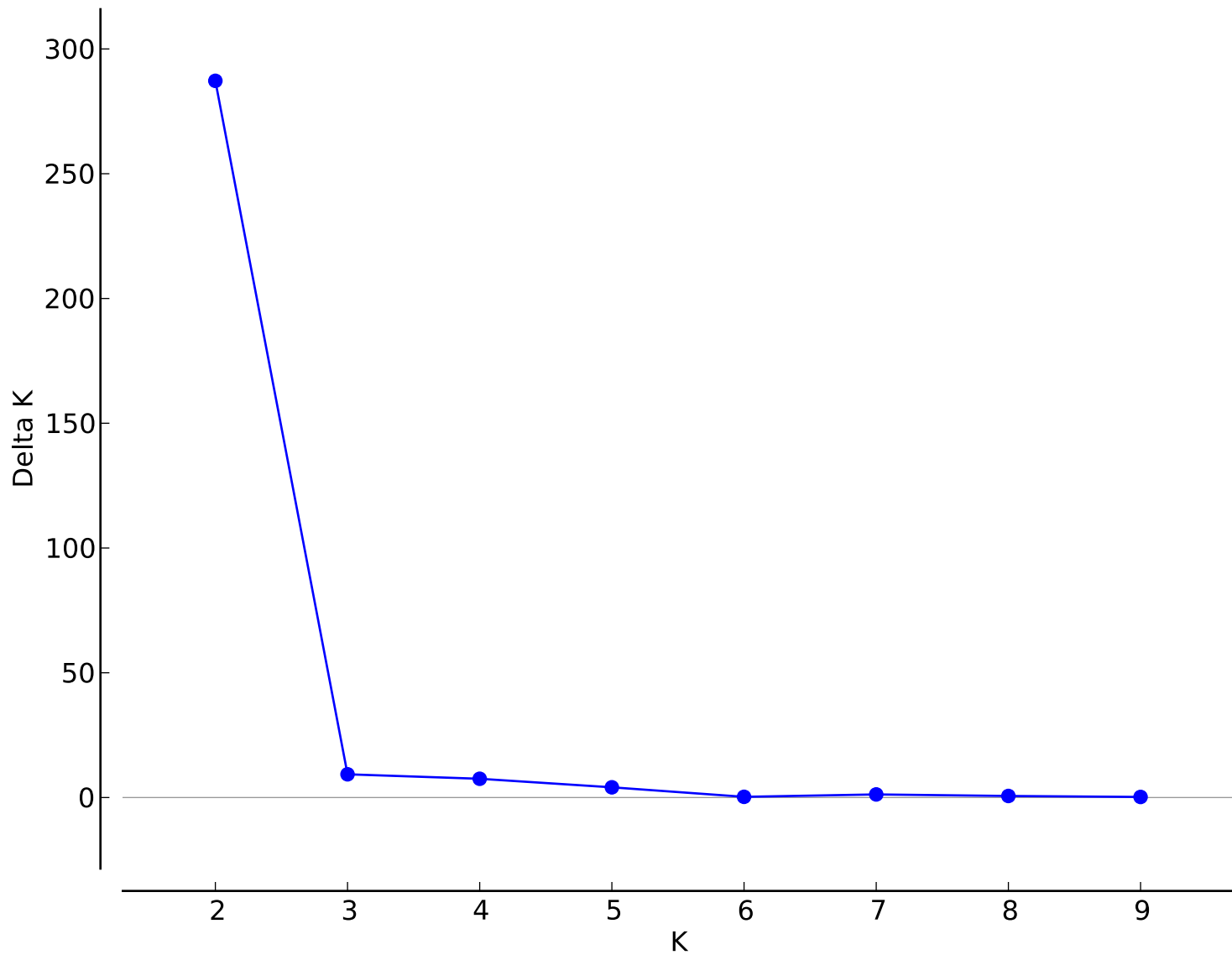

Supplement: Figure S1 [file peerj-06-4649-s001.pdf]

# Value of BIC versus number of clusters

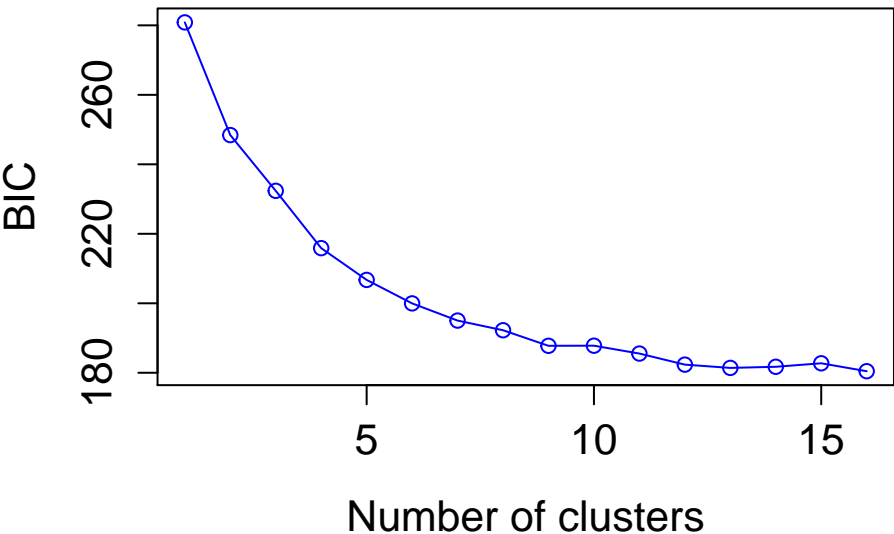

Supplement: Figure S3 [file peerj-06-4649-s003.pdf]
