# Supplementary material for: Living upside down: patterns of red coral settlement in a cave
Source: PeerJ. 2018 May 21;6:e4649. doi: 10.7717/peerj.4649 (PMC5967367; doi:10.7717/peerj.4649)
Supplement: Figure S2 [file peerj-06-4649-s002.pdf]

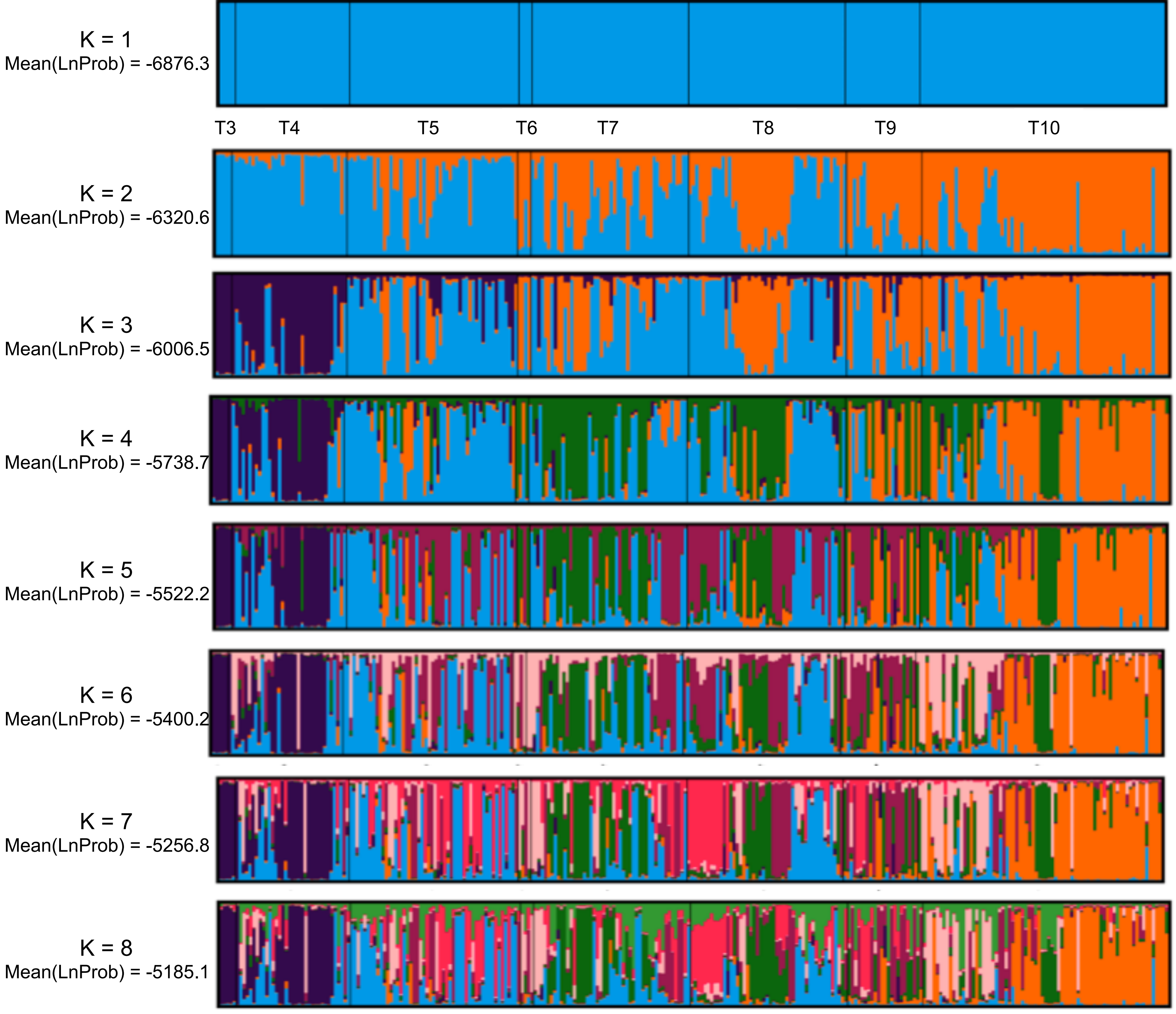
K = 1
Mean(LnProb) = -6876.3
T3
T4
T5
T6
T7
T8
T9
T10
K = 2
Mean(LnProb) = -6320.6
K = 3
Mean(LnProb) = -6006.5
K = 4
Mean(LnProb) = -5738.7
K = 5
Mean(LnProb) = -5522.2
K = 6
Mean(LnProb) = -5400.2
K = 7
Mean(LnProb) = -5256.8
K = 8
Mean(LnProb) = -5185.1
